# Characteristics of longitudinal maternal health studies in sub‐Saharan Africa: A systematic mapping of literature between 2012 and 2022

**DOI:** 10.1002/ijgo.16035

**Published:** 2024-11-16

**Authors:** Ijeoma Solarin, Cherlynn Dumbura, Darshnika Pemi Lakhoo, Kshama Chande, Gloria Maimela, Stanley Luchters, Matthew Chersich, for the HEAT Center

**Affiliations:** ^1^ Wits Planetary Health Research Division, Faculty of Health Sciences University of the Witwatersrand Johannesburg South Africa; ^2^ Centre for Sexual Health and HIV/AIDS Research (CeSHHAR) Harare Zimbabwe; ^3^ Wits RHI, Faculty of Health Sciences University of the Witwatersrand Johannesburg; ^4^ Department of International Public Health Liverpool School of Tropical Medicine (LSTM) Liverpool UK; ^5^ Department of Public Health and Primary Care Ghent University Ghent Belgium; ^6^ Public Health and Primary Care, School of Medicine Trinity College Dublin Ireland

**Keywords:** clinical trials, longitudinal studies, maternal health, prospective cohorts, research funding, sub‐Saharan Africa, systematic mapping

## Abstract

**Background:**

High maternal mortality rates in sub‐Saharan Africa necessitate the need for aligned research focusing on prevalent causes and neglected conditions in the region.

**Objective:**

This mapping review aimed to describe the characteristics of longitudinal maternal health studies between 2012 and 2022 in sub‐Saharan Africa and identify gaps in priority conditions or geographical locations.

**Search Strategy:**

We identified references through a Medline (PubMed) search covering September 2012 to June 2022.

**Selection Criteria:**

We included prospective cohort or clinical trials that enrolled at least 1000 pregnant women, with a study site in sub‐Saharan Africa, and published in English or French.

**Data Collection and Analysis:**

Screening and data extraction were done in duplicate using EPPI‐reviewer software. Descriptive analysis was used to summarize the results, identifying patterns in studies across time, country, study design, topics, and funders.

**Main Results:**

We identified 213 eligible studies, which were covered in 534 publications. We identified studies in 33 of the 48 sub‐Saharan African countries, with the majority in east and southern Africa. The predominant study topics were HIV (36.4%), nutrition (20%), and malaria (16.3%), with very few publications on hypertensive disorders of pregnancy (6.4%), ante/postpartum hemorrhage (3.7%), and sexually transmitted infections (3.2%). More studies were cohorts (115/213; 54%) than clinical trials. The National Institutes of Health (31.5%), Bill and Melinda Gates Foundation (22.1%), and USAID (10.8%) were the largest research funders.

**Conclusion:**

Identifying research trends and mismatches between research topics and disease burden provides useful information for guiding future research prioritization. In particular, gaps exist for studies on hypertensive disorders of pregnancy and ante/postpartum hemorrhage, among the top causes of maternal mortality in sub‐Saharan Africa.

## Synopsis

Mapping of maternal health studies in sub‐Saharan Africa reveals research gaps for important maternal mortality causes, including hypertensive disorders and ante/postpartum hemorrhage.

## 1 INTRODUCTION

Maternal deaths remain a major concern in sub‐Saharan Africa (sSA). Women in this region have a lifetime risk of dying due to pregnancy‐related complications of 1 in 42 versus 1 in 5300 in high‐income countries.^1^ The large majority of sSA countries are in danger of not meeting the United Nations Sustainable Development Goal of reducing global maternal mortality ratios (MMRs) to 70 per 100 000 live births by 2030.^2^ Average MMR in sSA currently stands at 531 per 100 000 live births^1^ with some countries such as Nigeria and South Sudan having MMR rates that are double this rate (Nigeria: 1047; Sierra Leone: 1223). sSA also has the highest rate of neonatal mortality with 27 deaths per 1000 live births compared with 2.7 deaths per 1000 live births seen in high‐income countries.^3^

The leading direct causes of maternal mortality in sSA are ante/postpartum hemorrhage, hypertensive disorders of pregnancy (HDP), sepsis and complications due to unsafe abortions, while leading indirect causes include HIV and malaria.^4^, ^5^, ^6^ Sexually transmitted infections (STIs) such as syphilis also pose significant burdens to maternal and neonatal health in the sub‐region.^7^ While there is general consensus around the leading causes of maternal deaths, it is important to assess whether current research trends align with these priorities. Understanding the research landscape helps to identify key neglected conditions and provides evidence to direct research priorities. Two previous reviews have tried to address this using different approaches. The first review, published in 2016 (review period: 2000–2012), focused on mapping research related to maternal health interventions for five key conditions: hemorrhage, hypertension, malaria, HIV, and other STIs.^8^ The second review, conducted in 2022 (review period: 2010–2019), concentrated on randomized trials in maternal and perinatal health.^9^ Both reviews were focused on low‐and middle‐income countries (LMICs), but not specifically on sSA. These studies both identified disparities in the geographical distribution of research publications as well as mismatches between topics covered and the causes of maternal deaths. There is a need for an updated review that focuses on sSA and specific health concerns in the sub‐continent.

We report the findings of a mapping review which examined the overall characteristics of longitudinal maternal health studies in sSA and describe the distribution of studies across countries, time, regions, funding sources, and disease burden.

## 2 METHODS

This review describes the outcomes of the systematic mapping of longitudinal maternal health studies in sSA identified in publications between 2012 and 2022. The review, building on a previous similar review,^8^ was done as a preliminary step to identify eligible studies for an individual participant data (IPD) meta‐analysis on heat exposure and maternal and child health. Participant‐level data from these studies will be linked temporally and spatially with climate and other environmental data. Analysis will define patterns of heat impacts in these populations and provide key information for informing heat‐health related responses to climate change in LMICs.^10^ The IPD falls within activities of the NIH HE^2^AT Center.^11^

A mapping review differs from classic systematic reviews which tend to address a single research question. Instead, it maps out the characteristics of studies on a broad topic area and identifies linkages and gaps in research.^8^, ^12^ This approach is thus highly suitable for summarizing the trends in maternal health research.

### 2.1 Search strategy and information sources

#### Identifying studies

Studies were identified through a database search that was first conducted on the October 10, 2020 in Medline (PubMed) and updated on the July 18, 2022 for articles published between September 1, 2012 and June 30, 2022. The search strategy (Appendix S2) was adapted from a previous review^8^ and involved controlled vocabulary and free‐text terms. We used search terms for maternal health which covered the ante‐ and postnatal periods and included biological or health system outcomes. We also applied validated search filters for locating cohorts and clinical trials, and for restricting studies to humans.^13^ sSA countries were as defined by the World Bank in 2019.^14^ The search terms for maternal health, cohort/trial study designs, and sSA countries were then combined using the AND Boolean term. There were no language restrictions applied in the search. We drew on the previous mapping review to identify additional references for the period January–August 2012.

#### Eligibility criteria

As this mapping review was conducted in the context of an IPD meta‐analysis, the inclusion/exclusion criteria had to consider the conceptual framework of the IPD. IPD meta‐analyses require a large amount of time and resources for data acquisition, preparation, and analysis of each individual study, and therefore only studies with enrolment of at least 1000 participants were included. In addition, considerations had to be made for the purpose of the larger study objective, which is to link data from the IPD to other environmental data. We therefore only included studies published between 2012 and 2022 as IPD and higher‐quality environmental data are more likely to be available. Other eligibility criteria included being a prospective cohort or clinical trial (CT) that enrolled women in pregnancy or intrapartum in a study site in sSA. Studies could be single‐ or multi‐site, and across one or more countries in Africa or other parts of the world. We excluded systematic and narrative reviews, reports of birth registries, cross‐sectional and case control studies, health and demographic surveillance data, and studies not in English or French.

#### Screening and data extraction

Screening of titles and abstracts for eligibility was done independently in duplicate, with differences between reviewers reconciled through discussion, or by a third reviewer. Abstracts in French were translated using Google Translate. We then screened potentially eligible full text articles and extracted the following study‐level data from the included publications: country, study design, topic, year of publication, and funders. We used EPPI‐Reviewer software for managing references, screening and data extraction.^15^

#### Deduplication

We conducted a deduplication process to identify publications linked to one main parent study. We matched studies by using a combination of any of the following where available: study name, clinical registration number, author/principal investigator details, study site, study period, funding details, enrolment number or other reported study identifiers.

#### Registration

The review protocol for the systematic search and identification of eligible studies is registered in PROSPERO [CRD42020214637].^16^ The IPD protocol has also been registered [CRD42022346068].^17^

### 2.2 Data analysis

Data checks were performed in the EPPI‐Reviewer software and in Stata 17 (StataCorp LP, College Station, TX, USA); the latter was used for analysis. Characteristics of the studies, including research topic and funder, were compared by study setting (country/region) and design. We also examined whether there was a relationship between the number of studies and MMR and infant mortality rate (IMR). Some variables, such as study funder or topic, have multiple responses as studies could be on multiple topics and funded by several entities. The total *n* for some variables may therefore exceed the number of studies.

As noted earlier, studies may result in several publications. We therefore analyzed results at both the publication and study levels. Publication‐level variables include country/region, year of publication, and study topic, while study‐level variables are country/region, study design, and funder (Appendices S3, S4).

Categorical variables are described using frequencies and percentages, and continuous variables with median and interquartile range (IQR). For continuous variables, the Spearman rank test was used to identify correlations between MMR and IMR and number of studies. As the search only included part of the year for 2022, we calculated an annualized figure for that year, and divided the time period into three categories (2012–2014, 2015–2019, and 2020–2022).

## 3 RESULTS

**FIGURE 1:**
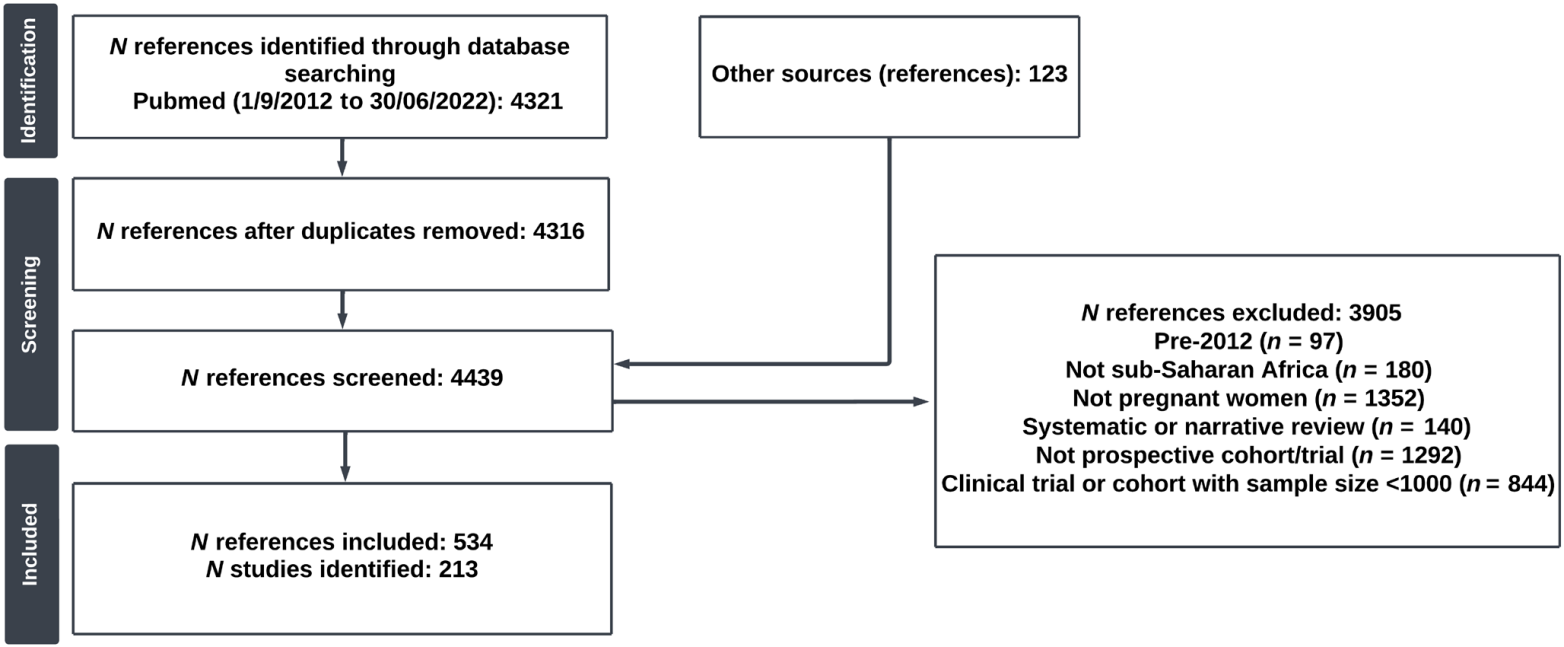
PRISMA flow diagram.

Of the 4439 publications identified through our search, 534 fulfilled eligibility based on full text. The most common reasons for exclusion were because they either did not enroll women in pregnancy (1352; 30.4%) or they were not longitudinal studies (1292; 29.1%). We also excluded 844 (19.0%) publications that were on longitudinal studies but reported enrolment numbers less than 1000. After matching publications to a parent study, we ended up with 213 individual studies (Figure 1).

**FIGURE 2:**
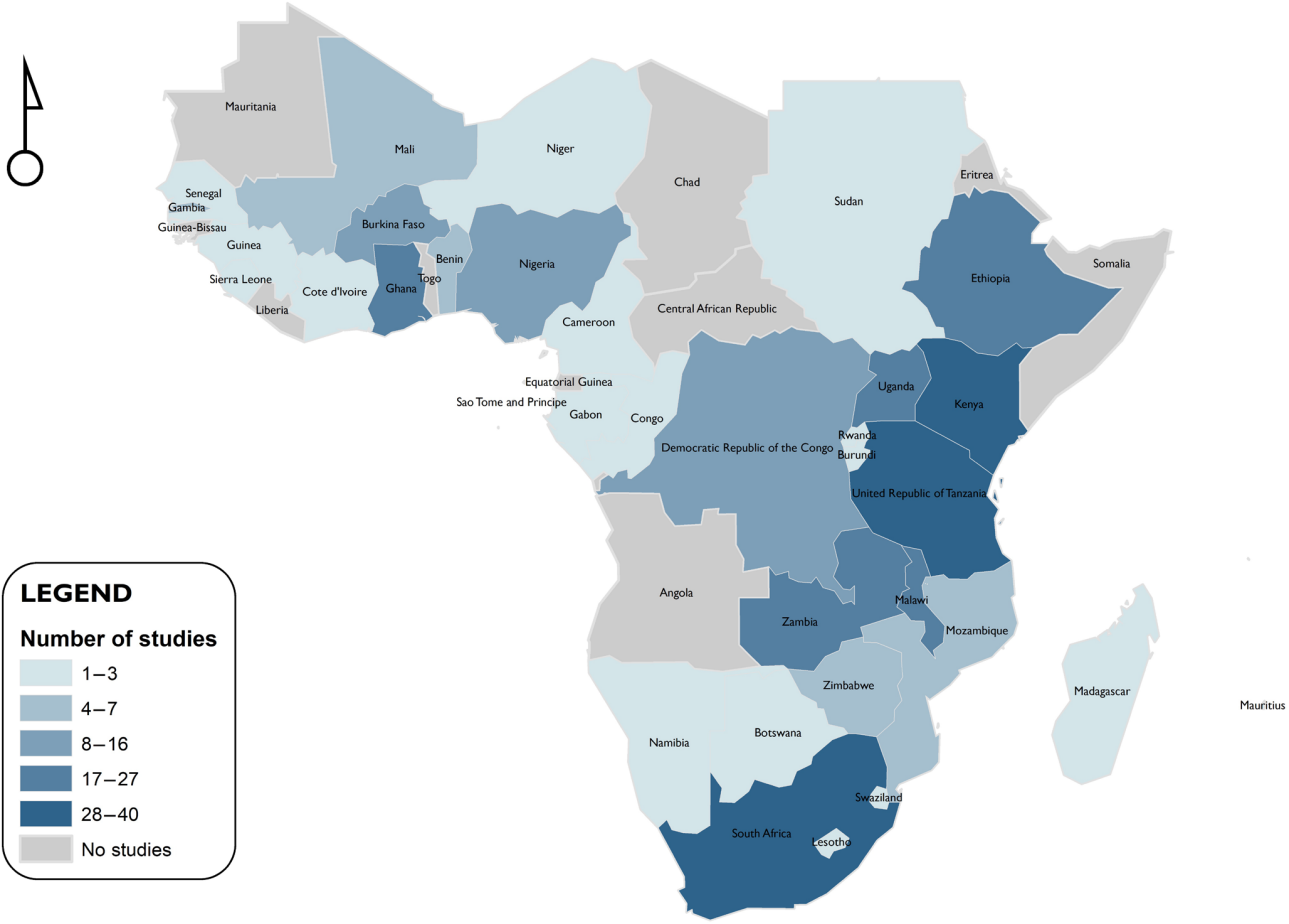
Map showing distribution of studies across the continent.

**FIGURE 3:**
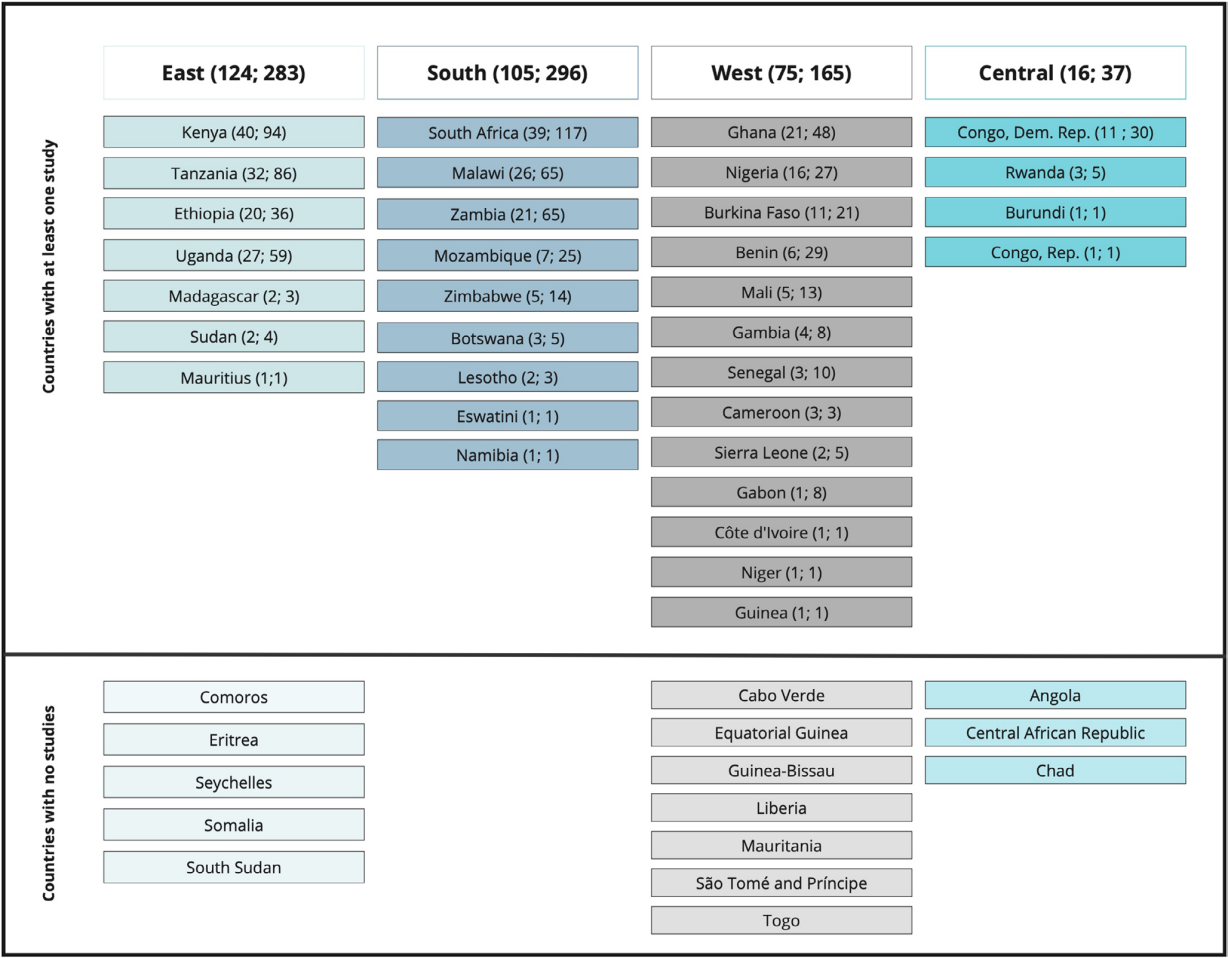
Regional distribution of studies in sub‐Saharan Africa (*n* studies; *n* publications). Regions are based on World Bank classification.

We identified studies in 33 of the 48 sSA countries (68.8%) and while there were studies in all regions, regional representation varied considerably. Southern Africa was fully represented with at least one study in all nine countries, while at the other extreme, in Central Africa we only identified studies in four out of seven countries (Figure 2). The southern African region had the greatest number of publications (*n* = 296, of which 117 (39.5%) were from South Africa). Central Africa had the least number of publications (*n* = 37), with the majority from the Democratic Republic of Congo (DRC) (81.1%) (Figure 3).

Of the 213 studies identified, 175 (82.2%) included only one sSA country site, while 38 (17.8%) included two or more sSA countries. The number of studies that were clinical trials (CT) (98; 46%) and cohorts (115; 54%) were similar overall and across regions (East: *n* = 124; 50% CT, 50% cohort; South: *n* = 105; 50.5% CT, 49.5% cohort; West: *n* = 75; 44% CT, 56% cohort) with the exception of Central Africa where there were almost twice as many cohorts as CTs (*n* = 16; 37.5% CT, 62.5% cohort). There were eight countries with 20 or more studies, with only three having more cohorts than CTs: Ethiopia (18 vs. 2), Ghana (13 vs. 8), and South Africa (27 vs. 12). As a whole, studies that included more than one sSA site had a higher proportion of CTs (*n* = 24; 63.2%) than cohorts (*n* = 14, 36.8%). We also observed that CTs produced more publications than cohort studies, with 3.1 papers per CT versus 2.2 publications per cohort study (*P* < 0.001).

South Africa had the highest number of publications (*n* = 117) followed by Kenya (*n* = 94). Most countries (19; 57.6%) had fewer than 10 publications (median 8; IQR 3–30) with 8 (24.2%) countries having only one publication (Burundi, Congo (Rep), Côte d'Ivoire, Eswatini, Guinea, Mauritius, Namibia, and Niger) and most of these were from one publication (*African Surgical Outcomes Study*).^18^ Most studies had resulted in only one publication (111, 52.1%) while 81 (38.0%) had between two and five publications, and 20 (9.4%) had more than five publications.

### 3.1 Publication trends

**FIGURE 4:**
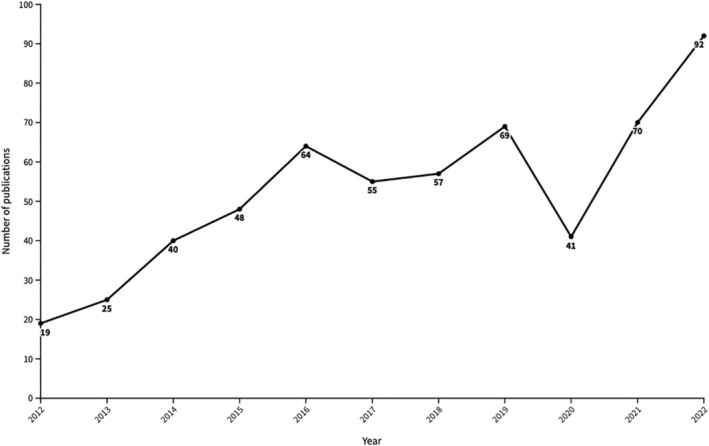
Annual number of publications.

We observed that the number of publications per year increased over the review period, particularly between 2012 and 2016, with a considerable decline in publications in 2020 (Figure 4). While Central Africa had the least number of publications, it had the largest increase in annual publications compared with the other regions, rising by seven‐fold between the period of 2012–2014 and 2020–2022. Only West Africa showed a noticeable decrease in publications between 2015–2019 and 2020–2022.

### 3.2 Topic trends

Publications covered a range of health topics and each publication could cover multiple topics. For this study, we report on the leading direct and indirect causes of maternal mortality in sSA, including HIV, malaria, HDP, and ante/postpartum hemorrhage as well as emergency obstetric care, nutrition, mental health, environmental exposures, and other sexually transmitted infections. Overall, HIV was the most published topic with 196 publications (36.4%), followed by studies on nutrition (108; 20.0%) and malaria (88; 16.3%). Altogether, these three topics were addressed in 312 (57.9%) publications. There were also differences between countries and regions. Almost half of the publications from studies conducted in Southern Africa were on HIV (46.3%), including all publications from Botswana (*n* = 5), Lesotho(*n* = 3), and Eswatini (*n* = 1), and proportions were likewise high in Zimbabwe (9; 64.3%), South Africa (66; 56.4%), and Malawi (32; 49.2%). By contrast, malaria was a predominant topic in West African countries: Gabon (8; 100%), Benin (27; 93.1%), The Gambia (5; 62.5%), Mali (4; 50%), and Burkina Faso (14; 66.7%). However, we did not identify any malaria studies in Nigeria, a country with one of the highest malaria burdens. Despite being leading causes of maternal mortality in sSA, we only identified 34 (6.4%) publications on HDP and 20 (3.7%) on hemorrhage. A further 41 publications covered composite outcomes of pregnancy, including studies on maternal morbidity, mortality, and emergency obstetric care. Publications on HPDs were spread across 13 countries, mostly covering East, West and Southern Africa (Mozambique [*n* = 8], Ethiopia [*n* = 6], Ghana [*n* = 6], South Africa [*n* = 6], Zambia [*n* = 6], Nigeria [*n* = 5], Uganda [*n* = 4], Zimbabwe [*n* = 4], Malawi [*n* = 3], Sierra Leone [*n* = 3], Tanzania [*n* = 2] and Kenya [*n* = 1]), with only one in Central Africa where MMR are highest globally. The greatest number of publications on hemorrhage were from Uganda (*n* = 8), Zambia (*n* = 7), Kenya (*n* = 6), and Ethiopia (*n* = 6). There were 47 publications on mental health, and most were from South Africa (38.3%) and Ethiopia (21.3%). Publications covering nutrition (*n* = 108) include both infant feeding and nutritional supplementation and were mostly from studies in Tanzania (38.9%) and Malawi (23.1%). Interestingly, we did not identify any studies investigating the effects of climate change on maternal health. We did, however, identify 16 publications on environmental exposures, with the majority centered around air pollution (*n* = 11; 68.8%) and primarily from Ghana (*n* = 7; 43.8%) and South Africa (*n* = 5; 31.3%).

**FIGURE 5:**
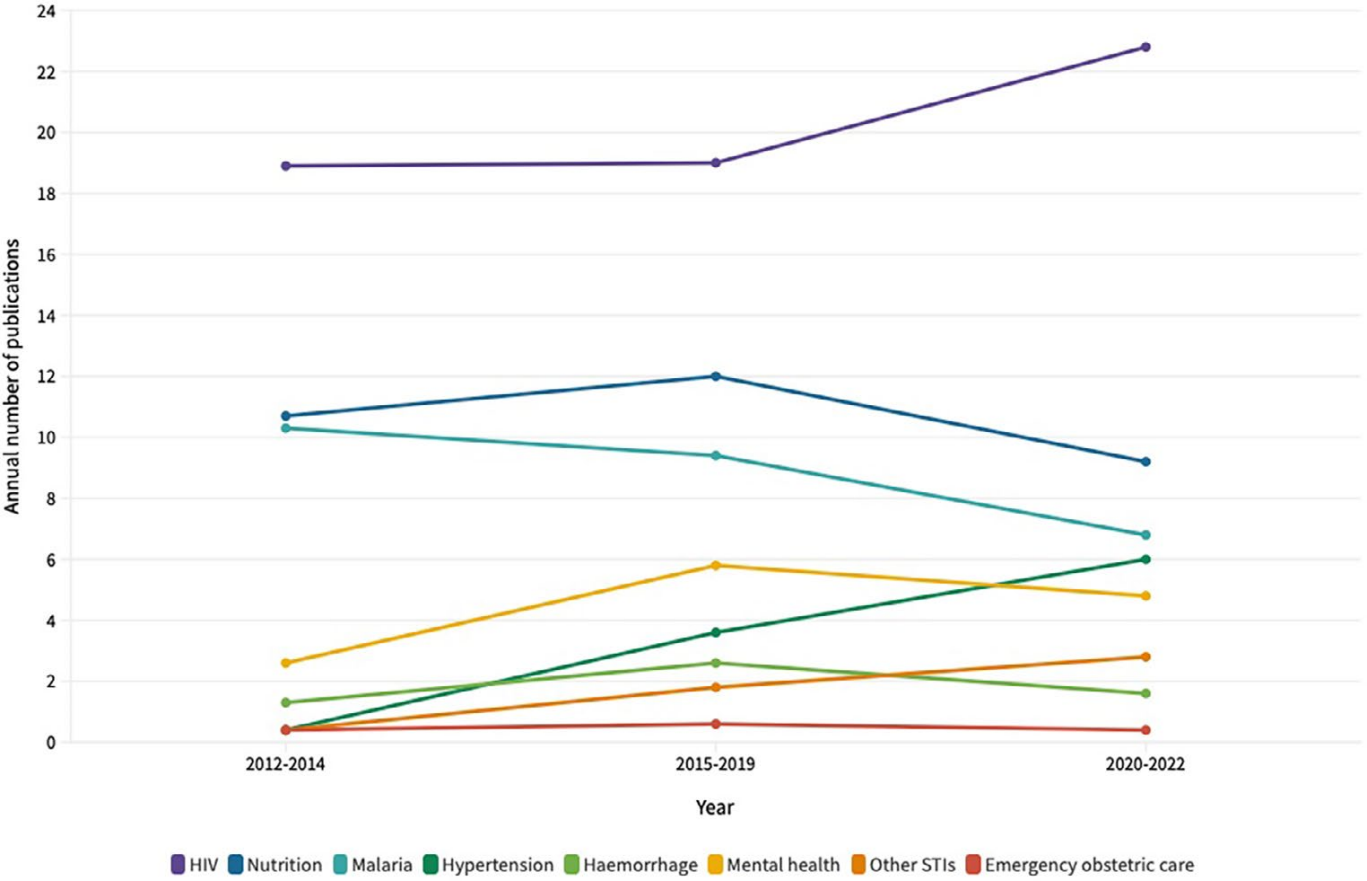
Annualized number of publications by topic in 3‐year group periods. Multiple response studies can include more than one topic. Publication numbers are annualized over the year group periods (number of studies divided by number of years or part of years).

When comparing the periods of 2012–2014 with 2020–2022, we observed a 15‐fold increase in annual publications on HDP. We also observed increases in HIV, other STIs, and mental health and a decrease in malaria and nutrition research output. Annual publications on hemorrhage, although increasing slightly in the 2015–2019 period, decreased in 2020–2022 (Figure 5).

We conducted a Spearman's rank correlation to assess the relationship between the number of studies and both the MMR and the IMR. The results indicated no significant correlation for either MMR (*r*_*s*_ = −0.13, *P* = 0.478) or IMR (*r*_*s*_ = −0.16, *P* = 0.381). Table 1 below outlines the characteristics of studies by region.

**TABLE 1:** Characteristics by region.

Characteristic	Total	East	South	West	Central
Number of studies^a^ (*n*, row%^b^)	213	124 (58.2%)	105 (49.2%)	75 (35.2%)	16 (7.5%)
*N* publications^a^ (*n*, row%^c^)	534	283 (52.9%)	296 (54.4%)	165 (30.8%)	37 (6.9%)
Annual publications^d^					
2012–2014	36	21.9	15.4	12	0.9
2015–2019	58.6	29.8	35.4	20	3.8
2020–2022	62.8	33.2	33.2	14	6.4
Study design (*n*, col.%^b^)					
Clinical trial	98 (46.0%)	62 (50%)	53 (50.5%)	33 (44.0%)	6 (37.5%)
Cohort	115 (54.0%)	62 (50%)	52 (49.5%)	42 (56.0%)	10 (62.5%)
Topics^a^ (*n*, col.%^c^)					
HIV/MTCT	196 (36.7%)	74 (26.1%)	137 (46.3%)	14 (8.5%)	3 (8.1%)
Nutrition	108 (20.2%)	58 (20.5%)	58 (19.6%)	19 (11.5%)	0
Malaria	88 (16.4%)	48 (17.0%)	41 (13.9%)	71 (43.0%)	1 (2.7%)
Mental health	47 (8.8%)	17 (6.0%)	25 (8.4%)	9 (5.5%)	1 (2.7%)
Hypertensive disorders of pregnancy	34 (6.4%)	13 (4.6%)	27 (9.1%)	14 (8.5%)	1 (2.7%)
Ante/postpartum hemorrhage	20 (3.7%)	26 (9.2%)	15 (5.1%)	13 (7.9%)	3 (8.1%)
STIs (other than HIV)	17 (3.2%)	7 (2.5%)	7 (2.4%)	2 (1.2%)	2 (5.4%)
Emergency obstetric care	5 (0.9%)	2 (0.7%)	2 (0.7%)	0	2 (5.4%)

Abbreviation: MTCT, Mother‐to‐Child Transmission; STI, sexually transmitted infection.

^a^ Multiple‐response categories.

^b^ Proportions are based on total number of studies (213).

^c^ Proportions are based on total number of publications (534).

^d^ Annualized number of publications for each time period (*n* publications/*n* years).

### 3.3 Funding trends

**FIGURE 6:**
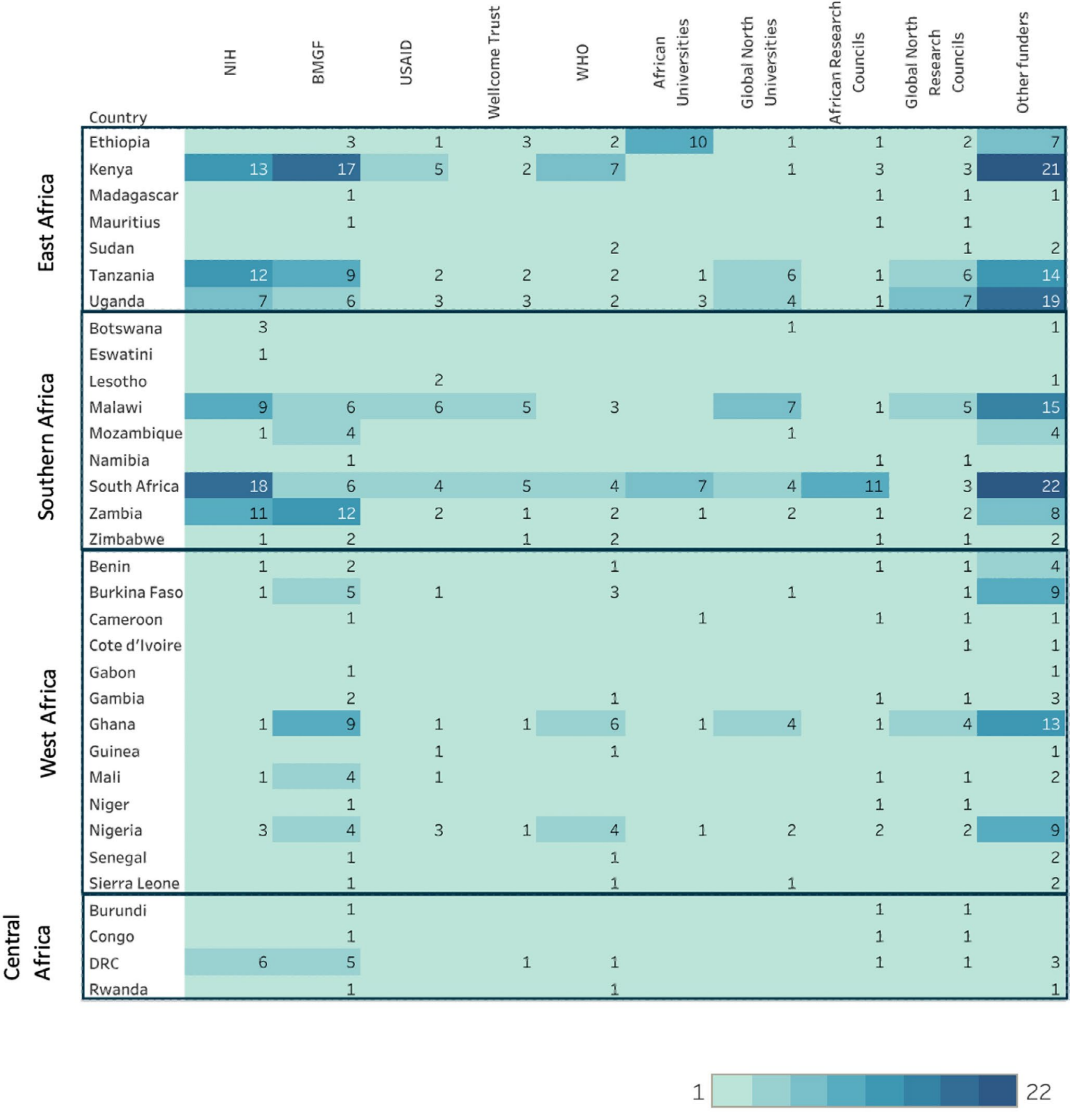
Heat map of funding allocation by country (n = number of studies).

We identified 176 different funding organizations providing support to studies which ranged from direct funding to providing assistance for publications, as well as training or fellowship grants. The top five identified funders were the National Institutes of Health (NIH) (67; 31.5%), Bill and Melinda Gates Foundation (BMGF) (47; 22.1%), USAID (23; 10.8%), Wellcome Trust (21; 9.9%), and WHO (18; 8.5%). Altogether, these five funder groups provided support for 137 (64.3%) studies. The largest number of studies funded by these organizations were found in Kenya (*n* = 44) and South Africa (*n* = 37). While the NIH supported the greatest number of studies, the BMGF supported studies in the greatest number of countries (27; 81.8%) (Table 2; Figure 6).

**TABLE 2:** Description of studies by funder.

Characteristic^b^	Total	NIH	BMGF	USAID	Wellcome Trust	WHO	Global North universities	African Universities	Global North research councils	African research councils	Other funders
*N* countries (*n*, row%^b^)	33	16 (48.5%)	27 (81.8%)	13 (39.4%)	11 (33.3%)	19 (57.6%)	13 (39.4%)	8 (24.2%)	24 (72.7%)	21 (63.6%)	27 (81.8%)
*N* studies (*n*, row%^c^)	213	67 (31.5%)	47 (22.1%)	23 (10.8%)	21 (9.9%)	18 (8.5%)	29 (13.6%)	25 (11.7%)	20 (9.4%)	15 (7.0%)	118 (55.4%)
*N* publications (*n*, row%^d^)	534	280 (52.4%)	187 (35.0%)	60 (11.1%)	64 (11.2%)	52 (9.7%)	102 (19.1%)	59 (11.0%)	73 (13.7%)	70 (13.1%)	322 (60.3%)
Publications per study (*n* studies, col.%^c^)											
1 publication	111	19 (28.3%)	14 (29.8%)	13 (56.5%)	10 (47.6%)	5 (27.8%)	8 (27.6%)	16 (64.0%)	8 (40.0%)	5 (33.3%)	62 (52.5%)
2–5 publications	81	30 (44.7%)	25 (53.2%)	8 (34.8%)	9 (42.9%)	12 (66.7%)	15 (51.7%)	8 (32.0%)	8 (40.0%)	6 (40.0%)	43 (36.4%)
>5 publications	20	17 (25.4%)	8 (17.0%)	2 (8.7%)	2 (9.5%)	1 (5.6%)	6 (20.7%)	1 (4.0%)	4 (20.0%)	4 (26.7%)	13 (11.0%)
Region (*n* studies, col.%^c^)											
East		32 (46.4%)	37 (77.1%)	11 (47.8%)	10 (45.5%)	15 (83.3%)	12 (41.4%)	14 (56.0%)	21 (100%)	8 (53.3%)	64 (53.8%)
South		44 (65.2%)	31 (66.7%)	14 (60.9%)	12 (59.1%)	11 (61.1%)	15 (51.7%)	8 (32.0%)	12 (57.1%)	15 (100%)	53 (45.4%)
West		7 (13.0%)	31 (64.6%)	7 (30.4%)	2 (9.1%)	18 (100%)	8 (27.6%)	3 (12.0%)	13 (71.4%)	8 (53.3%)	48 (40.3%)
Central		6 (8.7%)	8 (16.7%)	0	1 (4.5%)	2 (11.1%)	0	0	3 (14.3%)	3 (20.0%)	4 (3.4%)
Study design (*n* studies, col.%^c^)											
Clinical trial		40 (60.8%)	29 (62.5%)	10 (43.5%)	9 (45.5%)	10 (55.6%)	18 (62.1%)	2 (8.0%)	14 (71.4%)	5 (33.3%)	53 (45.4%)
Cohort		27 (39.1%)	18 (37.5%)	13 (56.5%)	12 (54.6%)	8 (44.4%)	11 (37.9%)	23 (92.0%)	6 (28.6%)	10 (66.7%)	65 (54.6%)

*Note*: Some other main funders: UKMRC (*n* = 17), DFID (*n* = 13), EU (*n* = 14), UN (*n* = 12), PEPFAR (*n* = 11), CDC (*n* = 9), World Bank (*n* = 6). Global North, Europe, North America, Australia.

Abbreviation: BMGF, Bill and Melinda Gates Foundation.

^a^ Multiple‐response categories, studies could be funded any combination of funders and each country in a multi‐country study is counted in the appropriate region.

^b^ Proportions based on total number of countries.

^c^ Proportions based on total number of studies.

^d^ Proportions based on total number of publications.

**FIGURE 7:**
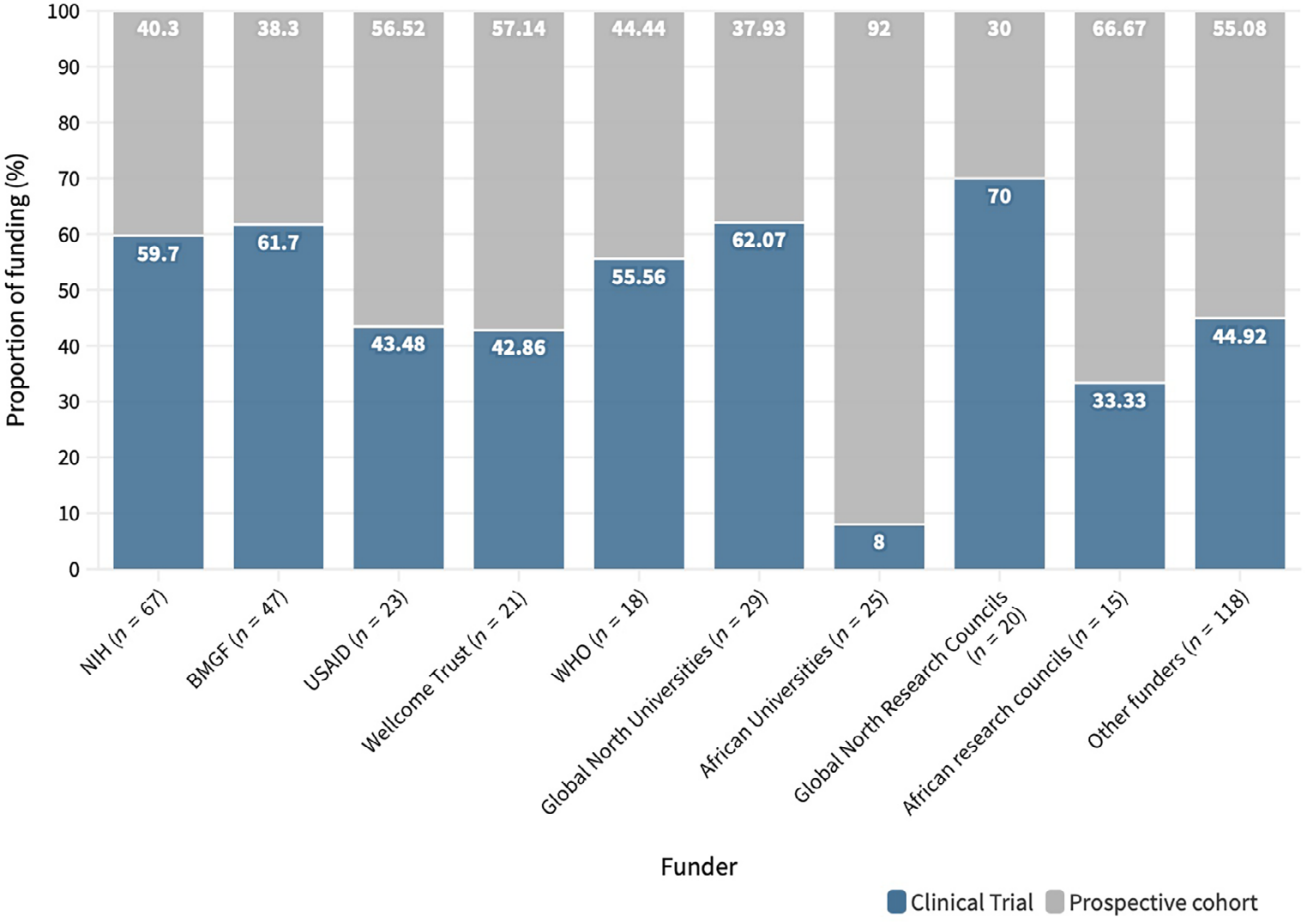
Proportion of funding allocated to cohorts and clinical trials by funder. BMGF, Bill and Melinda Gates Foundation; NIH, National Institutes of Health.

We also grouped funding by universities and research councils/institutes from the Global North and Africa. Global North universities and councils funded 29 (13.6%) and 20 (9.4%) studies, respectively, while for African universities and research councils the figures were 25 (11.7%) and 15 (7.0%). Of the 25 studies identified with funding from African universities, 14 were in East African countries and 10 of these were in Ethiopia. Similarly, of the 15 studies funded by African research councils, the majority went to studies in South Africa (11; 73.3%). We observed high levels of support for CTs from the NIH, BMGF, Global North universities, and councils while the opposite was true for African universities and research councils who overwhelmingly supported cohort designs (Figure 7).

## 4 DISCUSSION

This study describes patterns in research being carried out in sub‐Saharan Africa over the past 10 years (2012–2022) among pregnant women, a priority population for public health action. Over 4400 publications were screened to identify studies that would be eligible for an IPD analysis on the effects of heat on maternal and child health, which will be done once individual‐level data have been acquired and harmonized. Over 200 studies met the eligibility criteria covering 33 sSA countries.

Publications from these studies showed that there are marked disparities across the continent in terms of number of studies and topics, as well as funding. Of most concern, no studies were identified in 15 countries, some of which have the highest MMR on the continent such as the Central African Republic, Somalia, and South Sudan. Most of these countries have been involved in recent conflicts,^19^ which not only increases maternal mortality, but may also limit research operations. We also did not identify studies in some of the smaller countries such as Equatorial Guinea, Eritrea, Seychelles, and Sao Tome and Principe. These countries have less than 1 million women of reproductive age^20^ and therefore it may be more challenging to recruit participants into large studies in such settings. Countries with much larger population sizes such as Angola, Chad, and Niger also had no studies, suggesting that more is needed to build institutional and human resource capacity for research in these areas. In a recent publication, which mapped out research capacity within sSA countries, various indices were explored, such as number of publications, trials, universities, and researchers.^21^ The rankings largely correspond with our findings with most countries where we did not identify a study being ranked in the lowest percentile for research capacity to carry out large longitudinal studies. In addition to research capacity, poor national medicines regulatory authority (NMA) infrastructure may limit clinical trial research activities in many sSA countries. According to the WHO Global Benchmarking tool, only four countries in sSA have a NMA with a maturity level of 3 or 4 (reflects stable, well‐functioning, and integrated regulatory system), namely South Africa, Tanzania, Nigeria, and Ghana.^22^, ^23^ Increasing research and NMA capacity is imperative for providing much‐needed evidence to support improving maternal health outcomes. Populations across the sub‐continent are extremely diverse, limiting the ability to transfer evidence across countries, or even within countries.

There were noteworthy differences between regions. East and Southern Africa had the greatest number of studies and funding allocations. In West Africa, Ghana and Nigeria are prominent study sites, while the majority of studies from Central Africa were conducted in the DRC. This indicates that funders are partial to specific countries, which is then self‐perpetuating, where higher levels of funding enhances capacity and thus ability to publish findings and attract future funding.^24^ Indeed, a similar review noted that some research bodies tend to focus their funding on just a few countries.^21^ Funders may be unwilling to fund trials in countries with limited NMA infrastructure, as is the case in most sSA countries. We do, however, acknowledge that addressing maternal health extends far beyond research data and there may be valid reasons for more focus being placed on implementing programs in certain countries, rather than on research activities.

In line with findings from the previous review that covered the period 2000–2012,^8^ HIV and malaria made up a large proportion of the topics studied. HIV featured especially highly in publications from Southern Africa while malaria was predominant in publications from West African countries. This generally reflects the relative burden of disease in these regions,^25^ with some notable exceptions. Gabon, for example, has relatively low levels of malaria, but high research output on this topic. In contrast, while Nigeria has a very high malaria burden, with an estimated 25% of all global malaria cases,^26^ we did not identify any malaria studies from the country. This correlates with previous reviews and publications which have also noted mismatches between the leading causes of maternal mortality and research topics.^8^, ^9^ Importantly, although HDP and obstetric hemorrhage are leading causes of maternal mortality in sSA,^27^ there were relatively few publications identified on these topics. Other systematic reviews on maternal hemorrhage and HDP in sSA also demonstrate that there is a lack of longitudinal research in this area. In reviews on hemorrhage,^28^, ^29^ there were very few studies found and almost all were cross‐sectional in nature. While there were more studies found in reviews on HDP,^30^, ^31^ the study designs were mostly cross‐sectional or retrospective. Also, similar to the previous review,^8^ STIs continue to be under‐researched. There have been repeated calls for increased research into this important area,^32^, ^33^ given that the burden of curable STIs in pregnancy may be equal to or greater than that of malaria.^34^ It is promising that, unlike in the previous review, there was a continuous increase in STI research over time—a trajectory that needs to be maintained.

We acknowledge that our study had some limitations. We do not cover all of the longitudinal maternal health research conducted in sSA as we focused on studies with a sample size greater than 1000. This approach may be valid, however, as these larger sample sizes may provide greater power and generalizability, and thus ability to address priority questions in sufficient detail. In addition, our inclusion criteria were limited to studies in English or French, and so we may have missed studies done in less common languages on the continent. We also only searched one database (PubMed) and additional studies may have been identified if others were used. We did not assess the quality of the studies as this is outside the scope of a mapping review and will be done during the IPD analysis to follow. There is also a possibility that some publications from studies may have been missed if they were reporting on only a subset (*n* < 1000) of their data. Lastly, the team could have employed data science or bibliometric methods in the mapping. These methods may potentially reduce human error in screening and data extraction.

## 5 CONCLUSIONS

This mapping review provides important insights into maternal health research in sSA and for studies being used for the IPD analysis. Prioritizing research on maternal health is complex and resource constraints may mean that some countries as well as funders may place more focus on programmatic support, rather than research activities, depending on the needs and capacity of the country. Nevertheless, this review shows that there are few large longitudinal studies on the main causes of maternal mortality and other adverse maternal outcomes in sSA aside from HIV. In particular, there is a need for more robust longitudinal research on hemorrhage, HDP, and STIs. Additionally, research funding tends to be concentrated on a few countries. The gaps in geographical coverage make it challenging to generalize study finding across the sub‐continent, both in evidence reviews and in the planned IPD analysis.

## AUTHOR CONTRIBUTIONS

MFC and SL conceptualized the study. IS and MFC conducted the search. IS, CD, and KC screened and extracted data in Eppi‐Reviewer. IS and CD conducted the data analysis. IS prepared the original manuscript. IS, DL, MFC, SL, GM, CD, and KC reviewed and edited initial drafts. All authors were involved in critically reviewing and approving the final manuscript.

## FUNDING INFORMATION

Research reported in this publication was supported by the Fogarty International Center and National Institute of Environmental Health Sciences (NIEHS) and OD/Office of Strategic Coordination (OSC) of the National Institutes of Health under award number U54 TW 012083. The content is solely the responsibility of the authors and does not necessarily represent the official views of the National Institutes of Health.

## CONFLICT OF INTEREST STATEMENT

The authors have no conflicts of interest.

## Supporting information


**Appendix S1:** List of HE^2^AT Center group members.

**Appendix S2:** Full search strategy.

**Appendix S3:** Reference list of identified studies. This document lists all references identified in the search. Each reference has a publication number and an assigned study ID which is linked across all datasets.

**Appendix S4:** Study datasets. This is the study dataset containing two tables, one for the reference level analysis (S4a) and another for the study level analysis (S4b). Assigned study IDs and publication numbers link datasets and the reference list.

**Data S1:** Supporting Information.

## Data Availability

The data that supports the findings of this study are available in the supplementary material of this article.
