# Supplementary material for: Characteristics of longitudinal maternal health studies in sub‐Saharan Africa: A systematic mapping of literature between 2012 and 2022
Source: Int J Gynaecol Obstet. 2024 Nov 16;169(1):51–62. doi: 10.1002/ijgo.16035 (PMC11911973; doi:10.1002/ijgo.16035)
Supplement: Supplementary file 1 — Appendix S1: List of HE2AT Center group members. [file IJGO-169-51-s003.docx]

**Characteristics of longitudinal maternal health studies in sub-Saharan Africa: a systematic mapping of literature between 2012 and 2022**

**Appendix S1: List of HE^2^AT Centre Group Members**

CeSHHAR / Liverpool School of Tropical Medicine, Zimbabwe: Stanley Luchters (Principal Investigator), Cherlynn Dumbura, Jetina Tsvaki, Tatenda Makanga

IBM Research Africa: Sibusisiwe Makhanya (Co-Principal Investigator), Sibusisiwe Makhanya, Craig Mahlasi , Etienne Vos, Gciniwe Dlamini Baloyi, Tamara Govindasamy, Toby Kurien

Université Peleforo Gon Coulibaly, Ivory Coast: Guéladio Cissé (Co-Principal Investigator), Abdoulaye Tall, Adja Ferdinand Vanga, Brama Koné, Emmerence Okoue, Iba Diedonne Dely, Madina Doumbia, Yao Etienne Kouakou

University of Cape Town, South Africa (CSAG): Chris Jack (Co-Principal Investigator), Alice McClure, Lisa van Aardenne, Peter Marsh, Pierre Kloppers, Piotr Wolski, Rodger Duffett, Sabina Omar

University of Michigan, USA (CGHE): Akbar Waljee (Co-Investigator), Ji Zhu

University of the Witwatersrand, South Africa (Wits RHI): Matthew F Chersich (Principal Investigator), Craig Parker, Darshnika Pemi Lakhoo, Gloria Maimela, Ijeoma Solarin, Nicholas Brink, Relebohile Motana, Zororo Mavindidze

University of Washington, USA (CHANGE): Kris Ebi (Co-Principal Investigator), Chris Boyer

NIH: Bonnie Joubert, Kimberly McAllister, Maliha Ilias
