# Supplementary material for: Characteristics of longitudinal maternal health studies in sub‐Saharan Africa: A systematic mapping of literature between 2012 and 2022
Source: Int J Gynaecol Obstet. 2024 Nov 16;169(1):51–62. doi: 10.1002/ijgo.16035 (PMC11911973; doi:10.1002/ijgo.16035)
Supplement: Supplementary file 2 — Appendix S2: Full search strategy. [file IJGO-169-51-s005.docx]

**Characteristics of longitudinal maternal health studies in sub-Saharan Africa: a systematic mapping of literature between 2012 and 2022**

**Appendix S2: PubMed Search Strategy**

2012/09/01:2020/10/07[Date - Create] AND (("cohort studies"[MeSH Terms:noexp] OR "longitudinal studies"[MeSH Terms:noexp] OR "follow up studies"[MeSH Terms:noexp] OR "prospective studies"[MeSH Terms:noexp] OR "retrospective studies"[MeSH Terms:noexp] OR "cohort"[Title/Abstract] OR "longitudinal"[Title/Abstract] OR "prospective"[Title/Abstract] OR "retrospective"[Title/Abstract] OR ((("Clinical Trial"[Publication Type] OR "Clinical Trials as Topic"[MeSH Terms] OR "clinical trials"[All Fields]) AND "Clinical Trial"[Publication Type:noexp]) OR "clinical trial, phase i"[Publication Type] OR "clinical trial, phase ii"[Publication Type] OR "clinical trial, phase iii"[Publication Type] OR "clinical trial, phase iv"[Publication Type] OR "controlled clinical trial"[Publication Type] OR "multicenter study"[Publication Type] OR "randomized controlled trial"[Publication Type] OR "Clinical Trials as Topic"[MeSH Terms:noexp] OR "clinical trials, phase i as topic"[MeSH Terms:noexp] OR "clinical trials, phase ii as topic"[MeSH Terms:noexp] OR "clinical trials, phase iii as topic"[MeSH Terms:noexp] OR "clinical trials, phase iv as topic"[MeSH Terms:noexp] OR "controlled clinical trials as topic"[MeSH Terms:noexp] OR "randomized controlled trials as topic"[MeSH Terms:noexp] OR "early termination of clinical trials"[MeSH Terms:noexp] OR "multicenter studies as topic"[MeSH Terms:noexp] OR "Double-Blind Method"[MeSH Terms] OR (("randomised"[Title/Abstract] OR "randomized"[Title/Abstract]) AND ("trial"[Title/Abstract] OR "trials"[Title/Abstract])) OR (("single"[Title/Abstract] OR "double"[Title/Abstract] OR "doubled"[Title/Abstract] OR "triple"[Title/Abstract] OR "tripled"[Title/Abstract] OR "treble"[Title/Abstract] OR "treble"[Title/Abstract]) AND ("blind*"[Title/Abstract] OR "mask*"[Title/Abstract])) OR ("4 arm"[Title/Abstract] OR "four arm"[Title/Abstract]) OR "incidence"[MeSH Terms:noexp] OR "incidence"[Title/Abstract])) AND ("angola"[MeSH Terms] OR "benin"[MeSH Terms] OR "botswana"[MeSH Terms] OR "burkina faso"[MeSH Terms] OR "burundi"[MeSH Terms] OR "cabo verde"[MeSH Terms] OR "cameroon"[MeSH Terms] OR "central african republic"[MeSH Terms] OR "chad"[MeSH Terms] OR "comoros"[MeSH Terms] OR "democratic republic of the congo"[MeSH Terms] OR "congo"[MeSH Terms] OR "cote d ivoire"[MeSH Terms] OR "djibouti"[MeSH Terms] OR "equatorial guinea"[MeSH Terms] OR "eritrea"[MeSH Terms] OR "eswatini"[MeSH Terms] OR "ethiopia"[MeSH Terms] OR "gabon"[MeSH Terms] OR "gambia"[MeSH Terms] OR "ghana"[MeSH Terms] OR "guinea"[MeSH Terms] OR "guinea bissau"[MeSH Terms] OR "kenya"[MeSH Terms] OR "lesotho"[MeSH Terms] OR "madagascar"[MeSH Terms] OR "malawi"[MeSH Terms] OR "mali"[MeSH Terms] OR "mauritania"[MeSH Terms] OR "mozambique"[MeSH Terms] OR "namibia"[MeSH Terms] OR "niger"[MeSH Terms] OR "nigeria"[MeSH Terms] OR "rwanda"[MeSH Terms] OR "senegal"[MeSH Terms] OR "sierra leone"[MeSH Terms] OR "somalia"[MeSH Terms] OR "south africa"[MeSH Terms] OR "south sudan"[MeSH Terms] OR "sudan"[MeSH Terms] OR "tanzania"[MeSH Terms] OR "togo"[MeSH Terms] OR "uganda"[MeSH Terms] OR "zambia"[MeSH Terms] OR "zimbabwe"[MeSH Terms] OR "africa south of the sahara"[MeSH Terms] OR "africa, central"[MeSH Terms] OR "africa, southern"[MeSH Terms] OR "africa, eastern"[MeSH Terms] OR "africa, western"[MeSH Terms] OR ("angola"[Text Word] OR "benin"[Text Word] OR "botswana"[Text Word] OR "bechuanaland"[Text Word] OR "burkina faso"[Text Word] OR "burkina fasso"[Text Word] OR "upper volta"[Text Word] OR "burundi"[Text Word] OR "urundi"[Text Word] OR "cabo verde"[Text Word] OR "cape verde"[Text Word] OR "cameroon"[Text Word] OR "cameron"[Text Word] OR "cameroun"[Text Word] OR "central african republic"[Text Word] OR "ubangi shari"[Text Word] OR "chad"[Text Word] OR "congo"[Text Word] OR "zaire"[Text Word] OR "cote d ivoire"[Text Word] OR "cote d ivoire"[Text Word] OR "cote d ivoire"[Text Word] OR "ivory coast"[Text Word] OR "djibouti"[Text Word] OR "french somaliland"[Text Word] OR "equatorial guinea"[Text Word] OR "eritrea"[Text Word] OR "eswatini"[Text Word] OR "swaziland"[Text Word] OR "ethiopia"[Text Word] OR "gabon"[Text Word] OR "gabonese republic"[Text Word] OR "gambia"[Text Word] OR "ghana"[Text Word] OR "gold coast"[Text Word] OR "guinea"[Text Word] OR "kenya"[Text Word] OR "lesotho"[Text Word] OR "basutoland"[Text Word] OR "liberia"[Text Word] OR "madagascar"[Text Word] OR "malawi"[Text Word] OR "nyasaland"[Text Word] OR "mali"[Text Word] OR "mauritania"[Text Word] OR "mozambique"[Text Word] OR "portuguese east africa"[Text Word] OR "namibia"[Text Word] OR "niger"[Text Word] OR "nigeria"[Text Word] OR "rwanda"[Text Word] OR "ruanda"[Text Word] OR "senegal"[Text Word] OR "sierra leone"[Text Word] OR "somalia"[Text Word] OR "south africa"[Text Word] OR "south sudan"[Text Word] OR "sudan"[Text Word] OR "tanzania"[Text Word] OR "tanganyika"[Text Word] OR "togo"[Text Word] OR "togolese republic"[Text Word] OR "tonga"[Text Word] OR "uganda"[Text Word] OR "zambia"[Text Word] OR "zimbabwe"[Text Word] OR "northern rhodesia"[Text Word] OR "africa south of the sahara"[Text Word] OR "sub saharan africa"[Text Word] OR "subsaharan africa"[Text Word] OR "central africa"[Text Word] OR "sahara"[Text Word] OR "southern africa"[Text Word] OR "east africa"[Text Word] OR "eastern africa"[Text Word] OR "west africa"[Text Word] OR "western africa"[Text Word])) AND (("non-pregnancy"[All Fields] AND ("family"[MeSH Terms] OR "family"[All Fields] OR "relation"[All Fields] OR "relatability"[All Fields] OR "relatable"[All Fields] OR "related"[All Fields] OR "relates"[All Fields] OR "relating"[All Fields] OR "relational"[All Fields] OR "relations"[All Fields]) AND ("infections"[MeSH Terms] OR "infection"[All Fields] OR "communicable diseases"[MeSH Terms] OR ("communicable"[All Fields] AND "diseases"[All Fields]) OR "communicable diseases"[All Fields])) OR "non pregnancy related"[Title] OR (("maternal"[Title] OR "pregnant"[Title] OR "pregnancy"[Title] OR "obstetric"[Title] OR "puerperal"[Title] OR "mother"[Title] OR "childbirth"[Title] OR "labour"[Title] OR "labor"[Title] OR "natal"[Title] OR "post-natal"[Title] OR "pre-natal"[Title] OR "prenatal"[Title] OR "antenatal"[Title] OR "ante-natal"[Title] OR "perinatal"[Title] OR "peri-natal"[Title] OR "puerperal"[Title] OR "puerperium"[Title]) AND ("sepsis"[Title] OR "septic*"[Title] OR "infection*"[Title] OR "HIV"[Title] OR "tuberculosis"[Title] OR "pneumonia"[Title] OR "meningitis"[Title])) OR ("chorioamnionitis"[Title/Abstract] OR "chorioamnionitis"[MeSH Terms]) OR (("sepsis"[MeSH Terms] OR "sepsis"[All Fields] OR "septic*"[All Fields] OR "infection*"[Title]) AND ("amniotic"[Title/Abstract] OR "intra-amniotic"[Title/Abstract] OR "intraamniotic"[Title/Abstract])) OR (("anemic"[Title] OR "anaemia"[Title] OR "anaemic"[Title] OR "anemia"[Title]) AND ("puerperal"[Title] OR ("maternal"[Title] OR "pregnant"[Title] OR "pregnancy"[Title] OR "obstetric"[Title] OR "mother"[Title] OR "childbirth"[Title]))) OR ("Midwifery"[MeSH Terms] OR "dula"[Title/Abstract] OR ((("parturition"[MeSH Terms] OR "parturition"[All Fields] OR "birth"[All Fields]) AND ("attend"[All Fields] OR "attendance"[All Fields] OR "attendances"[All Fields] OR "attendant"[All Fields] OR "attendant s"[All Fields] OR "attendants"[All Fields] OR "attended"[All Fields] OR "attendence"[All Fields] OR "attendents"[All Fields] OR "attender"[All Fields] OR "attenders"[All Fields] OR "attending"[All Fields] OR "attendings"[All Fields] OR "attends"[All Fields])) OR (("parturition"[MeSH Terms] OR "parturition"[All Fields] OR "birth"[All Fields]) AND ("attend"[All Fields] OR "attendance"[All Fields] OR "attendances"[All Fields] OR "attendant"[All Fields] OR "attendant s"[All Fields] OR "attendants"[All Fields] OR "attended"[All Fields] OR "attendence"[All Fields] OR "attendents"[All Fields] OR "attender"[All Fields] OR "attenders"[All Fields] OR "attending"[All Fields] OR "attendings"[All Fields] OR "attends"[All Fields]))) OR ("residence characteristics"[MeSH Terms] OR ("residence"[All Fields] AND "characteristics"[All Fields]) OR "residence characteristics"[All Fields] OR ("place"[All Fields] AND "birth"[All Fields]) OR "place of birth"[All Fields]) OR ("Birthing Centers"[MeSH Major Topic] OR "Delivery Rooms"[MeSH Major Topic] OR "delivery, obstetric/nursing"[MeSH Major Topic]) OR (("maternal"[Title] OR "pregnant"[Title] OR "pregnancy"[Title] OR "obstetric"[Title] OR "puerperal"[Title] OR "mother"[Title] OR "childbirth"[Title] OR "labour"[Title] OR "labor"[Title] OR "natal"[Title] OR "post-natal"[Title] OR "pre-natal"[Title] OR "prenatal"[Title] OR "antenatal"[Title] OR "ante-natal"[Title] OR "perinatal"[Title] OR "peri-natal"[Title] OR "puerperal"[Title] OR "puerperium"[Title]) AND ("Ambulances"[MeSH Terms] OR "Health Services Accessibility"[MeSH Terms] OR "Transportation of Patients"[MeSH Terms])) OR (("Travel"[MeSH Terms] OR "delivery of health care/organization and administration"[MeSH Major Topic]) AND ("maternal"[Title] OR "pregnant"[Title] OR "pregnancy"[Title] OR "obstetric"[Title] OR "puerperal"[Title] OR "mother"[Title] OR "childbirth"[Title] OR "labour"[Title] OR "labor"[Title] OR "natal"[Title] OR "post-natal"[Title] OR "pre-natal"[Title] OR "prenatal"[Title] OR "antenatal"[Title] OR "ante-natal"[Title] OR "perinatal"[Title] OR "peri-natal"[Title] OR "puerperal"[Title] OR "puerperium"[Title]))) OR ("ectopic pregnancy"[Title/Abstract] OR "pregnancy, ectopic"[MeSH Terms]) OR ("Postpartum Hemorrhage"[MeSH Terms] OR (("maternal"[Title] OR "pregnant"[Title] OR "pregnancy"[Title] OR "obstetric"[Title] OR "puerperal"[Title] OR "mother"[Title] OR "childbirth"[Title] OR "labour"[Title] OR "labor"[Title] OR "natal"[Title] OR "post-natal"[Title] OR "pre-natal"[Title] OR "prenatal"[Title] OR "antenatal"[Title] OR "ante-natal"[Title] OR "perinatal"[Title] OR "peri-natal"[Title]) AND ("Hemorrhage"[Title] OR "Haemorrhage"[Title])) OR ((("obstetric"[All Fields] OR "obstetrically"[All Fields] OR "obstetrics"[MeSH Terms] OR "obstetrics"[All Fields] OR "obstetrical"[All Fields]) AND ("Haemorrhage"[All Fields] OR "Hemorrhage"[MeSH Terms] OR "Hemorrhage"[All Fields])) OR "obstetric hemorrhage"[Title/Abstract] OR ("Postpartum Hemorrhage"[MeSH Terms] OR ("postpartum"[All Fields] AND "Hemorrhage"[All Fields]) OR "Postpartum Hemorrhage"[All Fields] OR ("post"[All Fields] AND "partum"[All Fields] AND "Hemorrhage"[All Fields]) OR "post partum hemorrhage"[All Fields]) OR ("Postpartum Hemorrhage"[MeSH Terms] OR ("postpartum"[All Fields] AND "Hemorrhage"[All Fields]) OR "Postpartum Hemorrhage"[All Fields] OR ("post"[All Fields] AND "partum"[All Fields] AND "Haemorrhage"[All Fields]) OR "post partum haemorrhage"[All Fields]) OR ("Postpartum Hemorrhage"[MeSH Terms] OR ("postpartum"[All Fields] AND "Hemorrhage"[All Fields]) OR "Postpartum Hemorrhage"[All Fields] OR ("post"[All Fields] AND "partum"[All Fields] AND "Hemorrhage"[All Fields]) OR "post partum hemorrhage"[All Fields]) OR ("Postpartum Hemorrhage"[MeSH Terms] OR ("postpartum"[All Fields] AND "Hemorrhage"[All Fields]) OR "Postpartum Hemorrhage"[All Fields] OR ("post"[All Fields] AND "partum"[All Fields] AND "Haemorrhage"[All Fields]) OR "post partum haemorrhage"[All Fields])) OR "obstetric hemorrhage"[Title/Abstract] OR "hypertension, pregnancy induced"[MeSH Terms] OR ("obstructed labor"[Title/Abstract] OR "obstructed labour"[Title/Abstract] OR ("obstetric fistula"[Title/Abstract] OR "obstetric fistulae"[Title/Abstract]) OR ("vaginal fistula"[MeSH Terms] OR "vesicovaginal fistula"[MeSH Terms]) OR ("Obstetric Labor Complications"[MeSH Terms] OR "obstetric labor, premature"[MeSH Terms])) OR (("maternal"[Title] OR "pregnant"[Title] OR "pregnancy"[Title] OR "obstetric"[Title] OR "puerperal"[Title] OR "mother"[Title] OR "childbirth"[Title] OR "labour"[Title] OR "labor"[Title] OR "natal"[Title] OR "post-natal"[Title] OR "pre-natal"[Title] OR "prenatal"[Title] OR "antenatal"[Title] OR "ante-natal"[Title] OR "perinatal"[Title] OR "peri-natal"[Title]) AND ("hypertension"[Title] OR "blood pressure"[Title]) AND ("eclampsia"[Title/Abstract] OR "preeclampsia"[Title/Abstract] OR "HELLP"[Title/Abstract] OR "eclampsia"[MeSH Terms] OR "pre-eclampsia"[MeSH Terms] OR "pre-eclampsia"[Title/Abstract]))) OR ("pregnancy complications, hematologic"[MeSH Terms] OR "Pregnancy in Adolescence"[MeSH Terms] OR "pregnancy complications, infectious"[MeSH Terms] OR "pregnancy complications, cardiovascular"[MeSH Terms] OR "Pregnancy Complications"[MeSH Terms] OR "pregnancy, prolonged"[MeSH Terms])))
